# New Consumption Cure

**Published:** 1870-02

**Authors:** 


### NEW CONSUMPTION CURE.

About one person out of six in the United States dies of disease of the lungs, hence every reader has a more or less personal interest in informing himself as to the general nature of the ailment and its remedies.

There is an establishment on the beautiful and romantic banks of the Rhine for the treatment of consumption, called "The Grape Cure." Patients have excellent quarters, plenty of fresh air, and go into the gardens every day, in grape time, with a little basket which they fill, under the watchful eye of the doctor, then retire to an arbor and leisurely suck the grapes. A fine orchestra enlivens the curing process with delightful music. This plan of treatment is much more rational than many of those in our country; such as drinking whisky, swallowing cod-liver oil by the gallon, and crowding the stomach with dead dogs' bones, burned and ground into powder and administered under the scientific name of "phosphates." Cats' bones will do just as well, or those of a horse, or an octogenarian rat.

The reader may remember what a *furore* was created in New York City a few years ago in reference to the cure of consumption by inhalation ; that is, by introducing a variety of gases, or medicated air, into the lungs to come into immediate contact with the diseased parts. This seemed so plausible an idea that both the people and public press were carried away with it, and the man who advertised the plan made a large amount of money in a very short time, which he divided very liberally with the newspapers, secular and religious, paying sometimes for a single advertisement, in one paper, over five hundred dollars. The articles were well written, and fortified with extensive quotations from the works of eminent medical men, reaching back half a century.

There were two considerations which the public seemed to have overlooked. If these men, who were foreigners, had the ability to cure consumption, why did they leave their own country where the disease was more prevalent than here, and where in a few years they could have amassed uncounted millions of money? Then again, if inhalation was so efficient in the cure of the terrible disease, and if it had been known for half a century, why was it that throughout the civilized globe there was not a physician of any school found to practice inhalation as a cure of consumption, except in Broadway, New York? In a short time the excitement passed away, as also did the inhalationists, and no one is so weak or so brave to-day, as to use inhalation as a means of cure, or to take money for advising it.

THE WHISKY CURE.

About the same time a rival consumption cure was established, the only means being the drinking of a large amount of spirits every day, washing in alcohol several times a day, and for some hours to inhale its fumes. For a season, large amounts of money were taken in, and long advertisements were put in the newspapers; but the people at length found that no one was cured, and everybody died who persisted in the treatment, and the place was broken up.

### FRESH AIR CURE.

The pure out-door air is the natural food of the lungs; when deprived of the required amount, they always become diseased; and not only is consumption ameliorated, but all diseases are benefited in proportion as the patient is able to breathe pure air.

### CONSUMPTION

Always comes on in one of two ways, the lungs not having fresh air enough supplied to them, or by not being able to take in enough, which latter condition is brought on in the first place by a sufficient amount of pure air not being supplied for weeks and months in succession.

Whenever a consumptive patient is brought to a physician, one symptom is always present, is never by any possibility absent in any one case; pain may be absent, cough may be absent, night sweats may be absent, and swollen feet and ankles also, but a want of breath is always present.

It is known how much air the lungs of a healthy man will hold; the proportions vary according to fixed, known physical conditions, depending on age, sex, and height. On measurement, the consumptive is always found to be able to take in a less amount of air than the body requires; this amount varies according to the advancement of the disease; in some, there is a deficit in the beginning of five per cent.; in others, twenty; in others, one-half the lungs are inoperative, are doing no good. This one thing must strike the thoughtful reader— if a quarter or half the lungs are useless, can take in no air, then the greater need that the air taken in should be the purest possible, which is out-door air; for there is no pure air inside of any four walls, even if the roof is taken off; hence it follows, that in the breathing of an out-door air, the natural food of the lungs, is absolutely indispensable in the amelioration of any case of actual consumption of the lungs; and those systems of treatment which accomplish this for the largest period of the daylight of every day, are to be most favored. This thought is brought to notice in this grape cure. And yet the practice of the people, excepting perhaps, one case in a hundred thousand, is to keep patients from getting a single breath of out-door air for weeks and even months at a time, for fear of their taking cold; yet, at the same time, they are observed while they are thus housed up, to be taking cold all the time; and always do die eventually. Every increase of cough is regarded as a fresh cold, and is looked upon as the death-knell of the patient, when, in reality, an increase of cough, which brings up phlegm more freely, is a step toward cure—toward emptying the lungs of that yellow matter, which, when not thus got rid of, is re-absorbed into the system, and causes night sweats, hectic, swollen ankles, and death.

### COUGH

In consumption is nature's instinctive effort to cure the disease; and yet, instead of promoting it, to enable us to get rid of what is killing us, we send to the drug store and inquire for something to cure the cough, to stop it and the simpleton's breath at the same time. One of the effects of out-door air is to soothe the cough by dissolving the phlegm, making it more liquid and more easily expectorated.

